# Development of ultrasound-based clinical, radiomics and deep learning fusion models for the diagnosis of benign and malignant soft tissue tumors

**DOI:** 10.3389/fonc.2024.1443029

**Published:** 2024-11-12

**Authors:** Xinpeng Dai, Haiyong Lu, Xinying Wang, Bingxin Zhao, Zongjie Liu, Tao Sun, Feng Gao, Peng Xie, Hong Yu, Xin Sui

**Affiliations:** ^1^ Third Hospital of Hebei Medical University, Shijiazhuang, China; ^2^ First Affiliated Hospital of Hebei North University, Zhangjiakou, Hebei, China; ^3^ Department of Pathology, The Third Hospital of Hebei Medical University, Shijiazhuang, Hebei, China; ^4^ Department of Nuclear Medicine, The Third Hospital of Hebei Medical University, Shijiazhuang, Hebei, China

**Keywords:** deep learning, fusion model, radiomics, soft tissue tumors, ultrasound

## Abstract

**Objectives:**

The aim of this study is to develop an ultrasound-based fusion model of clinical, radiomics and deep learning (CRDL) for accurate diagnosis of benign and malignant soft tissue tumors (STTs)

**Methods:**

In this retrospective study, ultrasound images and clinical data of patients with STTs from two hospitals were collected between January 2021 and December 2023. Radiomics features and deep learning features were extracted from the ultrasound images, and the optimal features were selected to construct fusion models using support vector machines. The predictive performance of the model was evaluated based on three aspects: discrimination, calibration and clinical usefulness. The DeLong test was used to compare whether there was a significant difference in AUC between the models. Finally, two radiologists who were unaware of the clinical information performed an independent diagnosis and a model-assisted diagnosis of the tumor to compare the performance of the two diagnoses.

**Results:**

A training cohort of 516 patients from Hospital-1 and an external validation cohort of 78 patients from Hospital-2 were included in the study. The Pre-FM CRDL showed the best performance in predicting STTs, with area under the curve (AUC) of 0.911 (95%CI: 0.894-0.928) and 0.948 (95%CI: 0.906-0.990) for training cohort and external validation cohort, respectively. The DeLong test showed that the Pre-FM CRDL significantly outperformed the clinical models (P< 0.05). In addition, the Pre-FM CRDL can improve the diagnostic accuracy of radiologists.

**Conclusion:**

This study demonstrates the high clinical applicability of the fusion model in the differential diagnosis of STTs.

## 1 Introduction

Soft tissue tumors (STTs) are a group of tumors originating from mesenchymal tissues with complex and varied histological presentations (1). Benign STTs are more prevalent, with an incidence rate of approximately 3 per 1,000 annually (2), whereas the annual incidence rate of soft tissue sarcomas is about 36 per million (3, 4). Both benign and malignant tumors can cause pain and discomfort due to their growth, with malignant tumors having a poor prognosis and low survival rates. Therefore, early and accurate diagnosis of these tumors is crucial. Traditional diagnostic methods rely on pathological examination, which often requires invasive biopsy, posing physical and psychological burdens on patients. Ultrasound, as a non-invasive imaging technique, is widely used for preliminary diagnosis and tracking of tumors due to its real-time capability, safety, and cost-effectiveness (5, 6). However, the interpretation of ultrasound images depends heavily on the clinician’s experience and knowledge, leading to subjectivity and potential diagnostic uncertainty (7).

In recent years, radiomics and deep learning (DL) have emerged as promising technologies in tumor research. radiomics can extract high-throughput quantitative features from the tumor to reveal its biological characteristics (8). Previous studies have used magnetic resonance imaging (MRI)-based radiomics to diagnose STTs and have achieved excellent performance in validation sets (9, 10). However, manually crafted radiomics features are often sensitive and low-level, possibly failing to fully characterize tumor heterogeneity (11). As a data-driven approach, DL can extract many quantitative, high-throughput features from medical images, aiding in diagnosis and prognosis (12). In a previous systematic review, Benjamin Wang et al. achieved an accuracy of 79% in diagnosing STTs using an ultrasound-based DL model, comparable to the performance of two radiology experts (13). In addition, Bin Long et al. applied a deep learning model to the differential diagnosis of five benign soft tissue tumors and soft tissue sarcoma, showing high sensitivity (14).

Currently, the combination of radiomics and DL provides a new research avenue to improve the performance of ultrasound diagnosis (15–17). Data fusion techniques, including feature fusion and decision fusion, reflect complementary information. Decision fusion combines independent decision results from multiple models or algorithms to form a final diagnostic decision (18). Feature fusion combines different types of features before constructing the model, enabling the use of more comprehensive features for training and exploring complex data associations to improve model generalization. Wang et al. achieved an AUC of 0.94 using an MRI-based radiomics nomogram for STTs diagnosis, outperforming individual radiomics features and clinical models (19). Li et al. accurately distinguished axillary lymph node status in breast cancer patients by constructing an MRI-based radiomics and DL fusion model (11).

To our knowledge, no studies have developed a fusion model to differentiate between benign and malignant STTs. Therefore, we aim to develop a fusion model that integrates clinical information, radiomics, and DL to improve the diagnostic accuracy of malignant STTs.

## 2 Materials and methods

### 2.1 Patients

**Figure 1:**
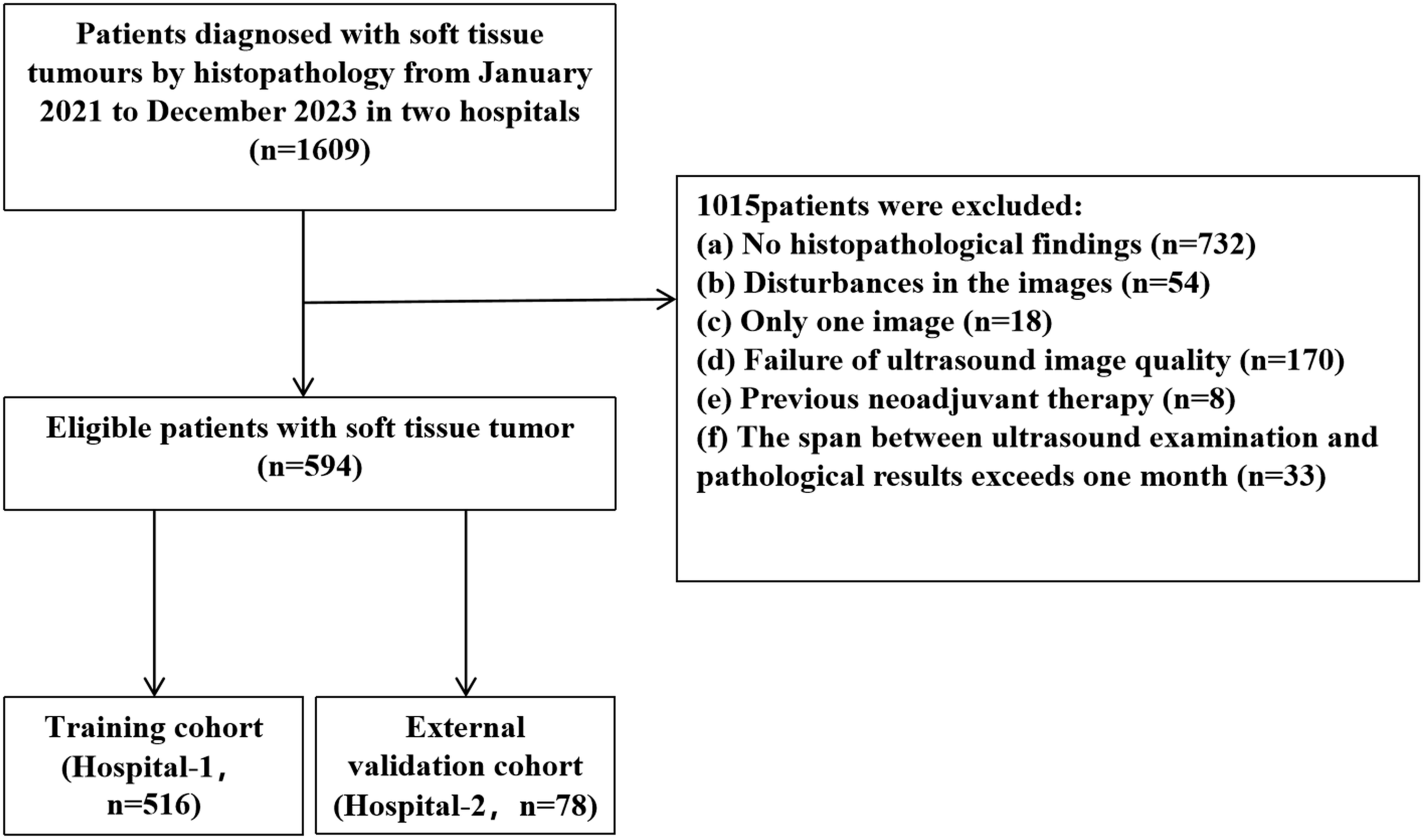
Patient selection flowchart.

In this study, we retrospectively collected data from 594 patients with superficial STTs at Hospital-1 and Hospital-2 between January 2021 and December 2023. Inclusion criteria were: (a) STTs confirmed by biopsy or surgery with complete pathological data; (b) images free of needle and foreign object interference; (c) ultrasound images including both 2D grayscale and color Doppler images; (d) clear images; (e) ultrasound examinations performed within one month before obtaining pathology results. Exclusion criteria were: (a) no histopathological findings; (b) interference by biopsy needles and other external objects; (c) prior neoadjuvant therapy. The patient recruitment flowchart is shown in **Figure 1**. Data from Hospital-1 served as the training cohort (TC), and data from Hospital-2 served as the independent external validation cohort (EVC). To dichotomize STTs, a small proportion of intermediate lesions (n = 11) were considered malignant for model training and evaluation. The study was approved by the Ethics Committee (approval number: KY2024-043-1) and informed consent was waived.

### 2.2 Clinical feature evaluation

Patient’s age and gender were extracted from the electronic medical record systems of the two hospitals. Routine semantic features of ultrasound were evaluated and extracted by two radiologists (with 10 and 20 years of experience, respectively) on the picture archiving and communication system (PACS) of the two hospitals. Ultrasound semantic features included maximum diameter, blood flow signal (0-1/2-3), morphology (regular/irregular), boundary (clear/unclear), and internal echo (uniform/uneven). If disagreement arises, the third radiologist is consulted to decide. A detailed description of the semantics is given in **Supplementary Table 1**.

### 2.3 Ultrasound imaging

Two images were selected for each patient, a greyscale image and a Doppler color image, which were used to train and evaluate the model. Images were acquired using 7-14 MHz linear array probes on HITACHI ALOKA3, Samsung HS70A, or PHILIPS HD154 systems under default instrument parameters. Images were exported and stored in digital imaging and communications (DICOM) format.

### 2.4 Analysis workflow

**Figure 2:**
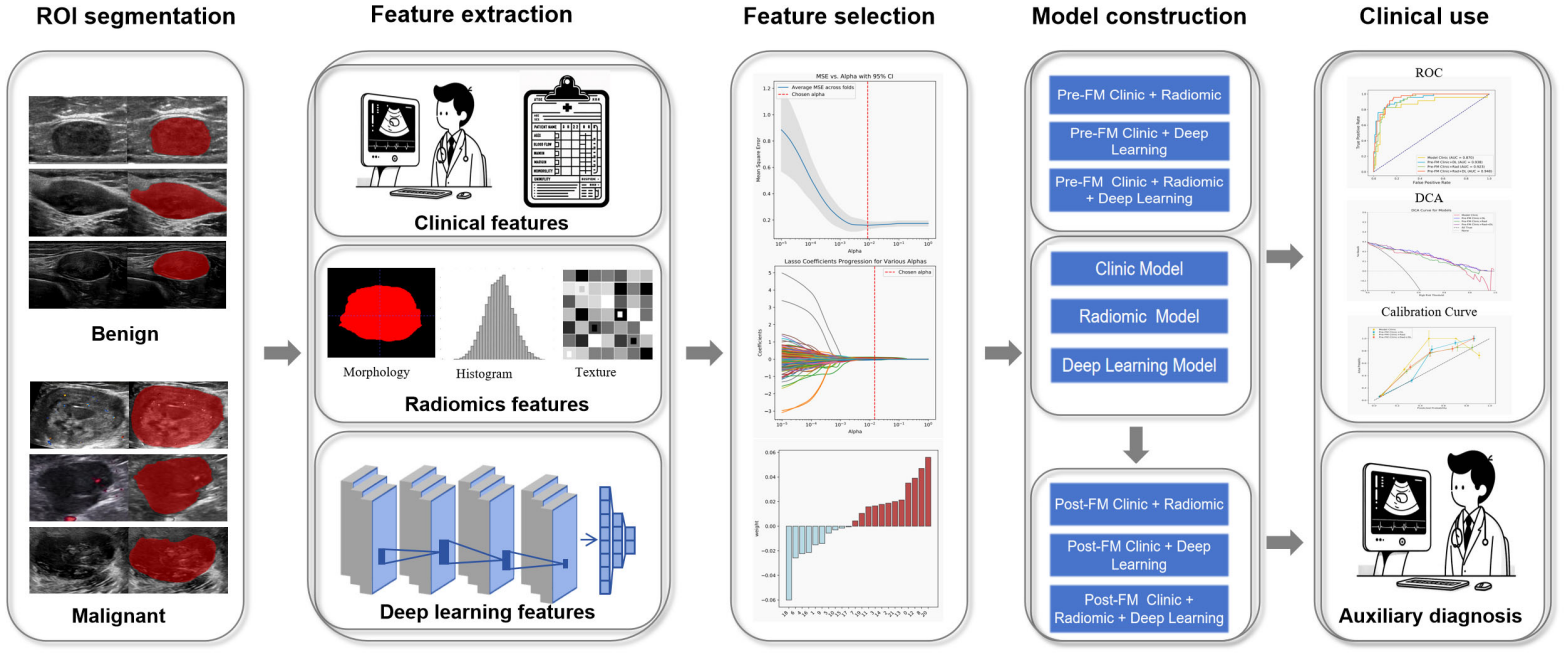
Workflow of model construction.

The workflow of this study includes region of interest (ROI) segmentation, clinical, radiomics, and deep learning feature extraction, and construction of pre-fusion and post-fusion models (**Figure 2**).

### 2.5 Image segmentation

To ensure data uniformity, a standardization process was implemented for all image data to eliminate possible intensity differences caused by different equipment and scan parameter settings. Image segmentation was performed independently by two radiologists who had no prior knowledge of the diagnostic histopathological findings. They performed the segmentation using the in-built region competition growth algorithm of the 3D-Slicer software (version 4.10.2, www.slicer.org) and performed a careful manual correction of the results. In case of disagreement, the decision was made in consultation with a third radiologist.

To evaluate feature stability, 50 patients were randomly selected from the training cohort after two weeks, and radiologists re-segmented the tumors to assess the inter- and intra-class correlation coefficients (ICC) between the extracted radiomics and DL features.

### 2.6 Signature extraction and construction

The open-source package “PyRadiomics” was leveraged to derive radiomics characteristics (20). In total, 851 radiomics features were extracted from the region of interest (ROI) for each patient. The handcrafted radiomics features included histogram, morphological, intensity, regularity, wavelet, and texture features. We adapted a 3D-ResNet to develop a deep convolutional neural network for DL feature extraction (15, 21). In our study, we employed the pre-trained 3D ResNet-18 model to automatically extract DL features from three-dimensional medical images. Initially, necessary preprocessing steps were applied to each image, including normalization and masking to remove non-interest areas. Specifically, we modified the ResNet model’s first layer to handle single-channel input and removed the final classification layer to extract feature vectors from the penultimate layer. A total of 511 DL features were extracted from the ROI of each patient. All features were normalized using the z-score method to standardize the values.

The extracted clinical, radiomics, and DL features will be combined to construct features for the pre-fusion model (Pre-FM). The Pre-FM features include clinical + radiomics features, clinical + DL features, and clinical + radiomics + DL features. To determine the correlation between the selected features, a Student’s t-test was used to screen and identify variables with significant discriminatory potential. Least Absolute Shrinkage and Selection Operator (LASSO) regression with 10-fold cross-validation was then used to select features that were highly correlated with identifying benign and malignant STTs.

Following the feature selection process, we proceeded to evaluate the stability of the selected features. To mitigate the variability across the TC and the EVC, the data underwent normalization through the z-score technique. The consistency of the classified semantic features extracted by different radiologists was critically assessed using Kappa statistics.

### 2.7 Model development

Four types of models were developed in this study: 1) Clinical Model (CM); 2) Single Modality Models (Rad-based and DL-based); 3) Pre-FMs (i.e., Clinic + Rad, Clinic + DL, and Clinic + Rad + DL [CRDL]); and 4) Post-fusion Models (Post-FMs, i.e., Clinic + Rad, Clinic + DL, and CRDL). Development of CM, single modal models, Pre-FM and Post-FM using Support Vector Machines (SVM) as classifiers. SVM is widely used in radiomics due to its efficient learning capability and has shown good performance in previous studies (22, 23). Data were split into training and internal validation cohorts on a TC. Using 5-fold cross-validation, 4/5 of the samples were randomly defined as the TC to train the model, and the remaining 1/5 were defined as the internal validation cohort to optimize parameters. This process was strictly repeated five times to obtain the optimal hyperparameter combination. Then, the model’s performance was tested on the EVC. Independence between the training and external validation data was strictly ensured to prevent data leakage. The optimal parameters for CRDL were: gamma, auto/kernel,and rbf. Other model parameters are provided in the **Supplementary Data 1.1** (**Supplementary Table A**). The model code is in the **Supplementary Data 1.2**.

### 2.8 Radiologist study

Two radiologists with different qualifications (Radiology A, 20 years of experience, and Radiology B, 5 years of experience) performed the diagnosis without knowledge of the pathological findings only on greyscale and Doppler images of 78 patients in the EVC. Second diagnosis was then made with the aid of the Pre-FM CRDL. A comparison of the radiologist’s diagnostic performance before and after the two diagnoses yielded the performance and clinical value of the Pre-FM CRDL.

### 2.9 Statistical analysis

Feature extraction, selection, model development, and validation were conducted using Python 3.7.1 (www.python.org). Statistical analyses were performed using SPSS software (version 25.0). Student’s t-test compared continuous variables and different models, while Pearson’s chi-square test or Fisher’s exact test compared categorical variables. The diagnostic performance of radiologists was compared using the McNemar test. Kappa statistics assessed the consistency of categorical semantic variables extracted by different radiologists. ICC was used to assess the consistency of continuous features extracted by radiologists. Model performance was evaluated using the area under the receiver operating characteristic (ROC) curve (AUC), accuracy (ACC), sensitivity, specificity, positive predictive value (PPV), and negative predictive value (NPV). DeLong’s test determined whether there were significant differences in AUCs between models (20). Calibration curves assessed the agreement between observed outcomes and the Pre-FM CRDL predictions. Decision curve analysis (DCA) quantified net benefit to evaluate the clinical usefulness of models in diagnosing benign and malignant STTs. A p-value less than 0.05 indicated statistical significance in all analyses.

## 3 Results

### 3.1 Clinical characteristics

**Figure 3:**
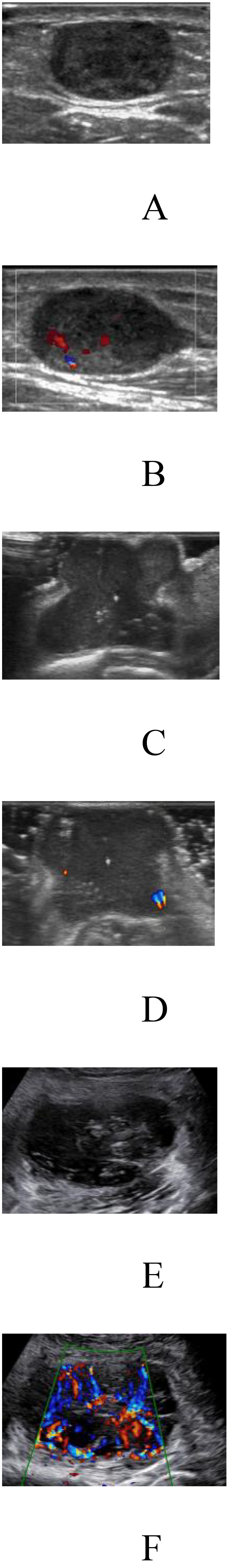
Two-dimensional grey-scale and color Doppler images of three patients with soft tissue tumors. **(A, B)** Images showing Schwannoma with regular morphology, well-defined borders, uneven internal echogenicity and blood flow grade 2. **(C, D)** Images showing trichoblastoma with irregular morphology, well-defined borders, uneven internal echogenicity and blood flow grade 1. **(E, F)** Images showing pleomorphic undifferentiated sarcoma with irregular morphology, poorly defined borders, uneven internal echogenicity and blood flow grade 3.

The clinical and imaging characteristics of the patients are shown in **Table 1**, and the distribution of the pathological findings is shown in **Supplementary Table 2**. Comparison of the relevant clinical and ultrasound characteristics of the patients with TC and EVC showed differences in age, tumor size and boundary. There was no statistically significant difference in the sample size of benign and malignant patients between the two groups, indicating a balanced subgroup sample size. The factors found to be significantly associated with the degree of malignancy of STTs by univariate and multivariate analyses are shown in (**Supplementary Table 3**). Patient age, tumor size, morphology, blood flow signal, and internal echo were independent predictors of the malignancy of STTs. **Figure 3** demonstrates 2D greyscale and color doppler images of Schwann cell tumor, trichoblastoma, and pleomorphic undifferentiated sarcoma. Malignant STTs tend to exhibit poorly defined borders and more abundant blood flow signals.

**Table 1:** Clinical features in the training and external validation cohorts.

Variable	Training cohort (n = 516)	External validation Cohort (n = 78)	*P* Value
Anthropometric			
Age	45.70 ± 18.89	52.35 ± 20.98	0.002
Sex			0.960
Female	246	38	
Male	270	40	
Pathological grading			0.101
Benign	410	55	
Malignant	106	23	
Semantic features			
Maximum diameter (mm)	4.15 ± 3.26	5.10 ± 3.26	0.005
Blood flow			
0-1	385	48	0.022
2-3	131	30	
Boundary (Margin)			<0.001
Clear	428	47	
Blur	88	31	
Morphology			0.339
Regular	311	52	
Irregular	205	26	
Uniformity			0.304
Yes	152	18	
No	364	60	

### 3.2 Feature stability

Both radiologists extracted ultrasound semantic variables with Kappa values greater than 0.80, indicating good agreement. The ICC of the radiomics and DL features extracted by two radiologists exceeded 0.80, indicating a high degree of consistency.

### 3.3 Radiomics and clinical features

Firstly, we constructed CM using five features: age, size, blood flow signal, morphology, and internal echo. Secondly, we developed single-modality models based on radiomics features and DL features. Next, we integrated clinical, radiomics, and DL features to build pre-fusion models, including Pre-FM Clinic + Rad, Pre-FM Clinic + DL, and Pre-FM CRDL. Finally, we developed three models by using SVM to fuse the probabilities from the respective model sets: Post-FM Clinic + Rad, Post-FM Clinic + DL, and Post-FM CRDL. The specific features and development parameters for all models are provided in **Supplementary Data 1.1**.

### 3.4 Model performance

**Figure 4:**
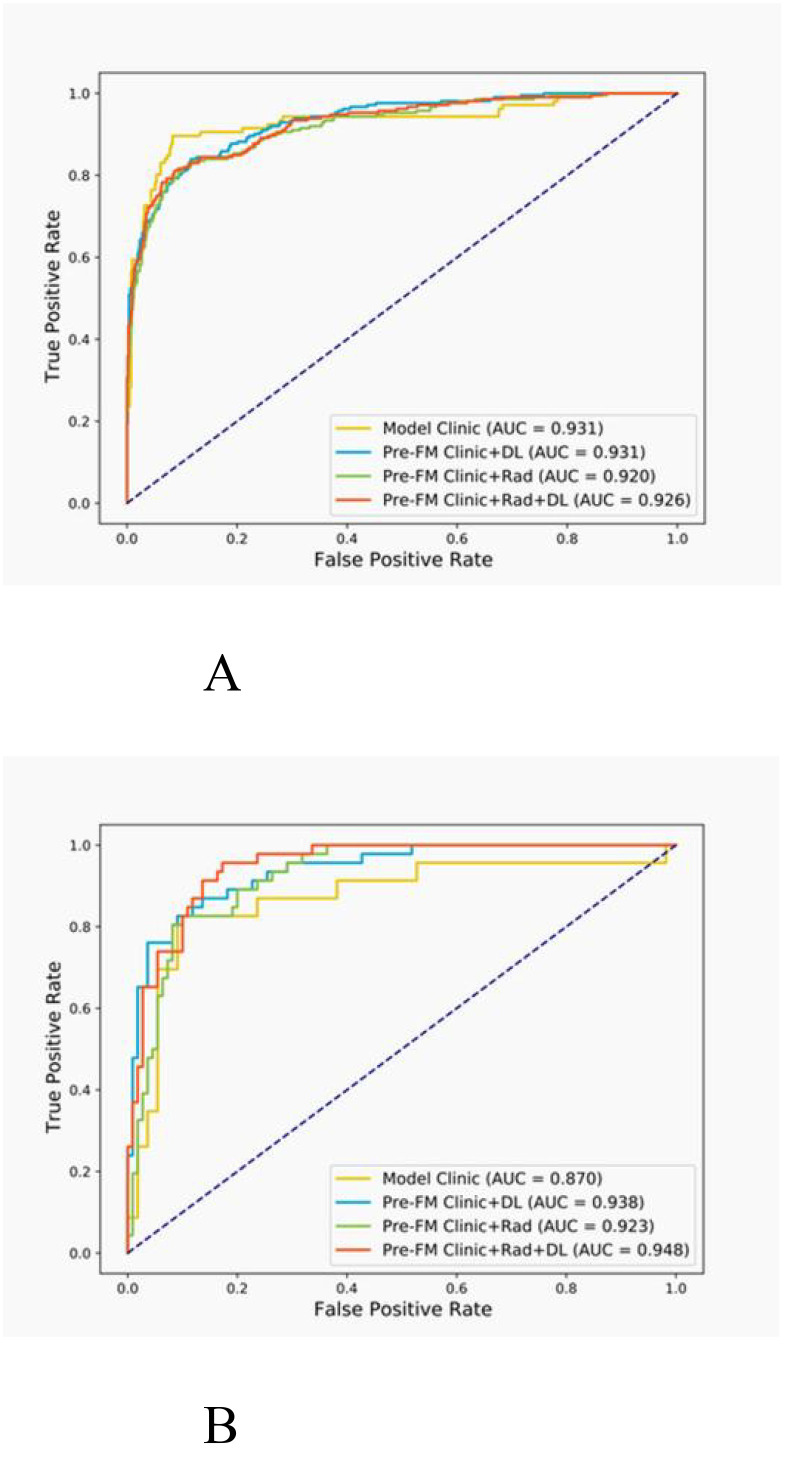
Receiver operating characteristic (ROC) curves for the pre-fusion model in the training **(A)** and external validation **(B)** cohorts.

Both the Pre-FMs and Post-FMs exhibited commendable accuracy in the diagnosis of STTs. In the preoperative diagnosis of STTs, Pre-FM CRDL demonstrated the best performance in the EVC (AUC 0.948, 95% CI 0.906-0.990). The ROC curves for Pre-FM in the training and EVC are shown in **Figures 4A, B**. The ROC curves for other models in the training and EVC are presented in **Supplementary Figures 1A–D**. Using a weighted Youden index to set the operating point, the sensitivity of the Pre-FM CRDL in the TC and EVC was 90.8% and 83.6%, respectively. Similarly, the specificity of Pre-FM CRDL in the TC and EVC was 81.0% and 89.3%, respectively. The statistical results for the CM and Pre-FM are presented in **Table 2**. Detailed statistical results for the single modality models and Post-FM are presented in **Supplementary Tables 4**, **5**.

**Table 2:** Performance of clinical models and pre-fusion models in training and external validation cohorts.

Group	Model	Auc	Sensitivity (%)	Specificity (%)	PPV (%)	NPV (%)	Accuracy (%)
TC	Model Clinic	0.916 (0.900-0.932)	91.9	81.7	96.1	67.5	90.2
	Pre-FM Clinic + Rad	0.911 (0.894-0.928)	90.1	82.2	96.7	59.0	89.0
	Pre-FM Clinic + DL	0.906 (0.890-0.922)	91.4	73.9	94.0	65.6	88.2
	Pre-FM CRDL	0.911 (0.894-0.928)	90.8	81.0	96.2	62.3	89.2
EVC	Model Clinic	0.870 (0.820-0.921)	88.1	84.2	94.5	69.6	87.2
	Pre-FM Clinic + Rad	0.923 (0.881-0.965)	81.4	81.5	95.5	47.8	81.4
	Pre-FM Clinic + DL	0.938 (0.897-0.980)	87.1	93.8	98.2	65.2	88.5
	Pre-FM CRDL	0.948 (0.906-0.990)	83.6	89.3	97.3	54.3	84.6

AUC, area under receiver operating characteristic curve; CRDL, clinical and radiomics and deep learning; DL, deep learning; EVC, external validation cohort; NPV, negative predictive value; PPV, positive predictive value; Pre-FM, pre-fusion model; Rad, radiomics; TC, training cohort.

The DeLong test was employed to determine if there was a significant difference in the AUC of the models in the two cohorts. As shown in **Table 3**, among Pre-FMs, the CRDL significantly outperformed CM (0.948 vs. 0.870, P = 0.01) and Clinic + Rad (0.948 vs. 0.923, P = 0.017). However, there was no significant difference in diagnostic performance between CRDL and Clinic + DL (0.948 vs. 0.938, P = 0.625). Among Post-FMs, CRDL, Clinic + Rad, and Clinic + DL could all distinguish benign from malignant STTs; however, there were no significant differences in diagnostic performance between models, as shown in **Supplementary Table 6**.

**Table 3:** Comparison of diagnostic performance between pre-fusion models.

Model vs. Model	P Value
TC	
Model Clinic vs. Pre-FM Clinic + Rad	0.463
Model Clinic vs. Pre-FM Clinic + DL	0.969
Model Clinic vs. Pre-FM CRDL	0.735
Pre-FM Clinic + Rad vs. Pre-FM Clinic + DL	0.106
Pre-FM Clinic + Rad vs. Pre-FM CRDL	0.044
Pre-FM Clinic + DL vs. Pre-FM CRDL	0.417
EVC	
Model Clinic vs. Pre-FM Clinic + Rad	0.108
Model Clinic vs. Pre-FM Clinic + DL	0.035
Model Clinic vs. Pre-FM CRDL	0.010
Pre-FM Clinic + Rad vs. Pre-FM Clinic + DL	0.450
Pre-FM Clinic + Rad vs. Pre-FM CRDL	0.017
Pre-FM Clinic + DL vs. Pre-FM CRDL	0.625

CRDL, clinical and radiomics and deep learning; DL, deep learning; EVC, external validation cohort; Pre-FM, pre-fusion model; Rad, radiomics; TC, training cohort.

**Figure 5:**
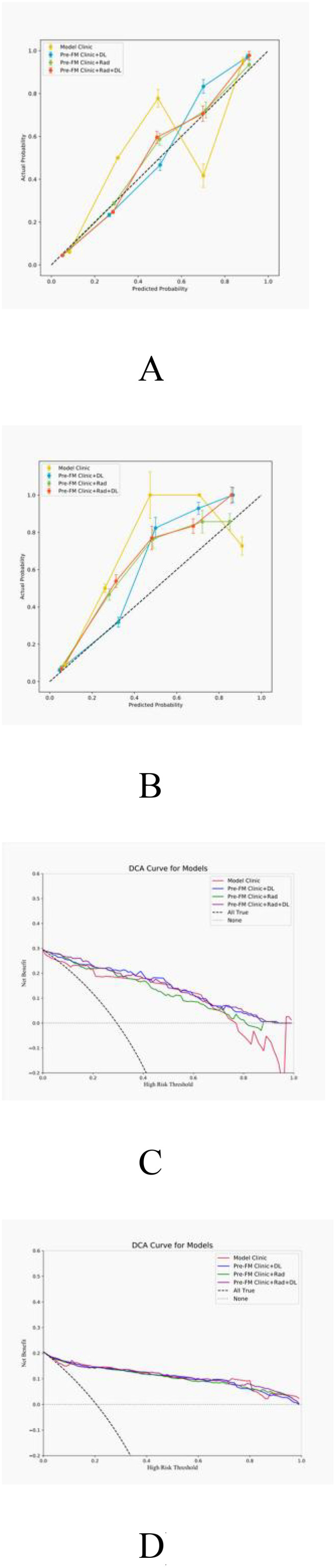
Calibration curves for pre-fusion models in the training **(A)** and external validation **(B)** cohorts; Decision curve analysis (DCA) of pre-fusion models in training **(C)** and external validation **(D)** cohorts.

The actual outcomes of STTs patients were consistent with the predictions of the Pre-FM CRDL in TC and EVC. The calibration curves of Pre-FMs in the two cohorts are shown in **Figures 5A, B**. Calibration curves for other models are presented in **Supplementary Figure 2**. The discriminatory power of these models was assessed using DCA to determine their clinical utility. The Pre-FM CRDL’s curve is higher than the other models at most risk thresholds, suggesting that it has better performance in predicting high risk. DCA for Pre-FM in TC and EVC is shown in **Figures 5C, D**. DCA for other models is presented in **Supplementary Figure 3**.

### 3.5 Radiologist study results

**Figure 6:**
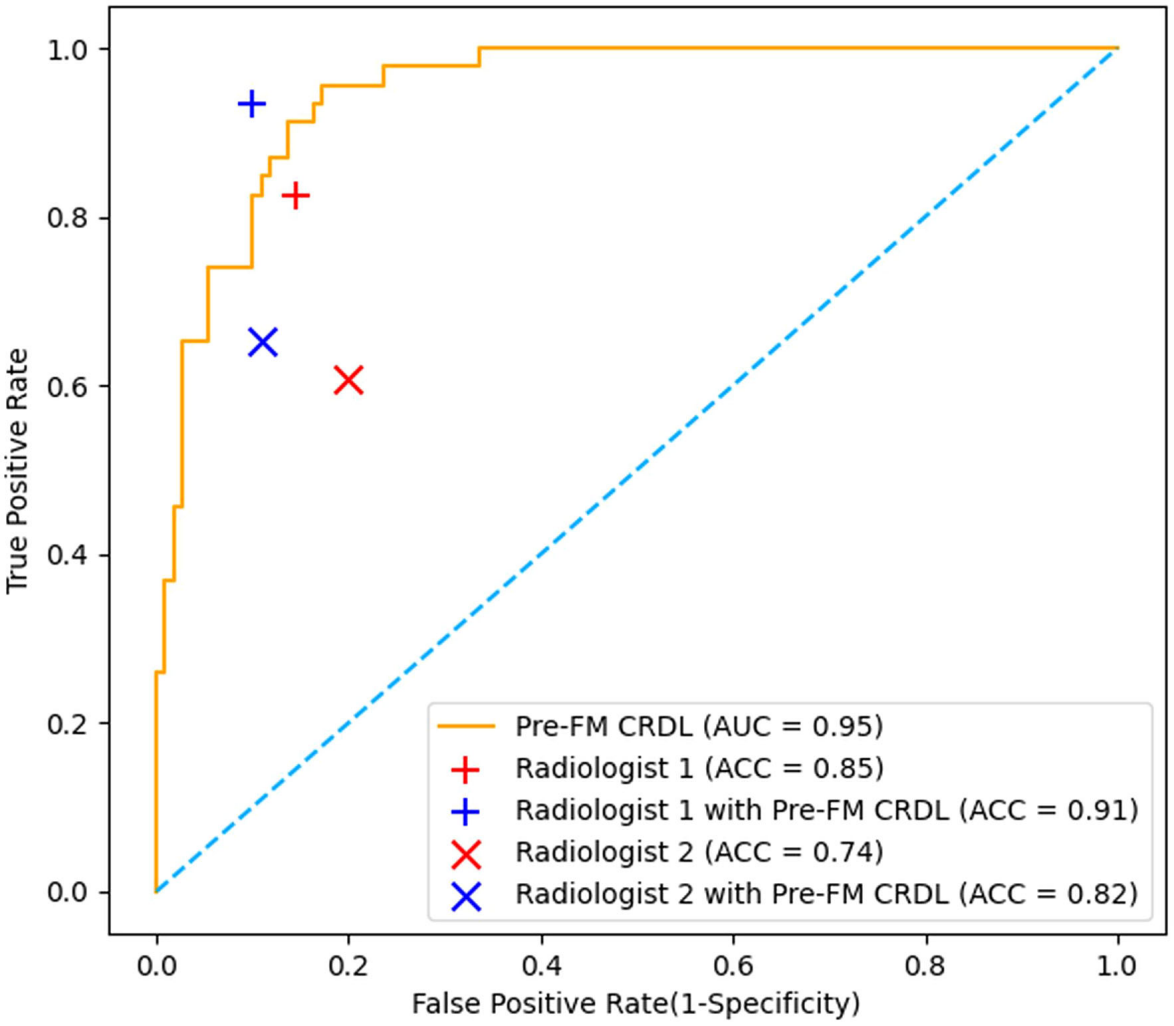
Comparison of Receiver operating characteristic (ROC) curves from the pre-fusion CRDL model with ROC curves from two radiologists.

Two musculoskeletal radiologists independently classified the external validation set and then reclassified with the assistance of Pre-FM CRDL. The results showed that Pre-FM CRDL improved the radiologists’ diagnostic accuracy. The younger radiologist reached the diagnostic level of the 20-year experienced radiologist with the model’s assistance. The ROC curves in **Figure 6** show the performance comparison between the model and the two radiologists. **Table 4** compares the sensitivity, specificity, PPV, NPV, and accuracy of the two radiologists with the model. McNemar’s test results indicated no significant differences between the radiologists’ independent diagnosis and model-assisted diagnosis results.

**Table 4:** Comparing radiologists’ performance in independent and model-assisted diagnosis.

Parameter	ACC (%)	Sensitivity (%)	Specificity (%)	PPV (%)	NPV (%)	P
Radiologist 1	84.6 (78.1-89.4)	82.6 (69.3-90.9)	85.5 (77.7-90.8)	70.4 (57.2-80.9)	92.2 (85.3-96.0)	0.79
Radiologist 1 with Pre-FM CRDL	91.0 (85.5-94.6)	93.5 (82.5-97.8)	90.0 (83.0-94.3)	79.6 (67.1-88.2)	97.1 (91.7-99.0)	
Radiologist 2	74.4 (67.0-80.6)	60.9 (46.5-73.6)	80.0 (71.6-86.4)	56.0 (42.3-68.8)	83.0 (74.7-89.0)	0.08
Radiologist 2 with Pre-FM CRDL	82.1 (75.3-87.3)	65.2 (50.8-77.3)	89.1 (81.9-93.6)	71.4 (56.4-82.8)	86.0 (78.4-91.2)	

CRDL, clinical and radiomics and deep learning; Pre-FM, pre-fusion model.

## 4 Discussion

In this study, we successfully developed three pre-fusion models based on feature level and three post-fusion models based on predictive probability for the diagnosis of benign and malignant STTs. Among these, the Pre-FM CRDL demonstrated high diagnostic accuracy (86.5%), excellent sensitivity (85.6%), and specificity (90.3%) by integrating clinical information, radiomics features, and DL features. Additionally, the Pre-FM CRDL improved the diagnostic accuracy of radiologists, indicating significant potential in differentiating benign from malignant tumors. As far as we know, this is the first study to use an ultrasound-based feature fusion model to predict the benign and malignant nature of STTs. Through innovative multi-feature fusion, it provides a new approach for the diagnosis of STTs.

Malignant STTs are characterized by irregular morphology, heterogeneous low echogenicity, and increased internal blood flow compared to benign tumors (24). Our multivariate analysis identified tumor size, morphology, blood flow signal, and internal echo as independent predictors of malignancy, aligning with traditional clinical experience and supporting the clinical value of radiomics in diagnosis (25). However, boundary clarity was not an independent predictor of malignancy, likely because most STTs, regardless of malignancy, have relatively clear margins. This finding is consistent with Hexiang Wang’s research, suggesting that traditional macroscopic imaging features might still have limitations in early malignant tumor diagnosis even with advanced imaging technologies (26). Fusion models that combine these macroscopic features extracted by radiologists with microscopic features provide a comprehensive diagnostic perspective, aiding clinicians in making more accurate treatment decisions.

Previous studies have shown that radiomics and DL models can predict the nature of STTs (14, 27, 28). Masataka Nakagawa et al. achieved an AUC of 0.89 using an MRI-based clinical information and radiomics fusion model for STTs diagnosis, outperforming individual radiomics features and clinical models (29). Long et al.’s ultrasound-based DL model, although performing well in the validation cohort, was limited by its generalization capability, only including diagnoses of five benign tumors (14). Moreover, despite its high sensitivity, their model’s specificity was below 50%, indicating a high rate of misdiagnosis. Benjamin Wang et al.’s study achieved 79% accuracy in the validation cohort but lacked external validation (13). In contrast, our Pre-FM CRDL not only exhibited high diagnostic accuracy but also balanced sensitivity and specificity, effectively reducing misdiagnosis and missed diagnosis, which is crucial for clinical decision-making.

Xie et al. developed a fusion model combining ultrasound-based deep learning features with clinical features (30). The model was able to significantly improve the diagnostic accuracy of soft tissue sarcomas by young radiologists in a prospective dataset. This result is similar to our study, suggesting that the fusion model has good potential for application in improving diagnosis. Our established Pre-FM CRDL model performed satisfactorily in clinical aid diagnosis. Specifically, the model enabled younger radiologists to achieve diagnostic levels comparable to those of 20-year experienced radiologists. Additionally, the misdiagnosis rate for malignant STTs significantly decreased with the model’s assistance, which is crucial for patient treatment and prognosis.

However, this study has some limitations. First, due to the diversity and rarity of STTs subtypes, this study cannot encompass all subtypes. Future research should increase sample size and further explore the characteristics of different subtypes. Second, the ultrasound images in this study were obtained from different devices and scanning parameters, which, while increasing the model’s robustness in real-world applications, may also introduce additional image heterogeneity, potentially affecting diagnostic accuracy and model stability (2, 19). Future research should consider standardizing the imaging acquisition process or developing advanced algorithms to mitigate device differences. Fourth, for some large STTs, the analysis may miss key radiomics and DL features due to the selection of non-maximal area complete 2D sections, potentially affecting diagnostic accuracy. Lastly, in this study, participants could only rely on pre-selected static 2D grayscale and color Doppler images for judgment. This design limitation might underestimate the actual diagnostic ability of radiologists. Finally, ultrasound contrast and elastography offer unique advantages in the diagnosis of STTs and may further improve diagnostic accuracy if these functional imaging are routinely performed in the future (31, 32).

## Conclusion

Compared to traditional radiomics and DL models, the ultrasound-based fusion model demonstrated superior performance in predicting benign and malignant STTs. Additionally, the fusion model provided clinical net benefits in DCA. Future studies should conduct international multicentre large sample studies to validate and optimise the diagnostic models with a view to achieving wider clinical applications and providing a scientific basis for individualised treatment of STTs.

## Data Availability

The raw data supporting the conclusions of this article will be made available by the authors, without undue reservation.
