# Supplementary material for: Development of ultrasound-based clinical, radiomics and deep learning fusion models for the diagnosis of benign and malignant soft tissue tumors
Source: Front Oncol. 2024 Nov 12;14:1443029. doi: 10.3389/fonc.2024.1443029 (PMC11588752; doi:10.3389/fonc.2024.1443029)
Supplement: Supplementary file 1 [file DataSheet1.docx]

Supplementary Material

Development of ultrasound-based clinical, radiomics and deep learning fusion models for the diagnosis of benign and malignant soft tissue tumors

Xinpeng Dai, Haiyong Lu, Xin Sui*****

*** Correspondence:**Xin Sui :38200395@hebmu.edu.cn

Department of Ultrasound, Hebei Medical University Third Hospital, Shijiazhuang, Hebei, China

# Supplementary Data

**1.1Model feature set and model parameter**

**CM:**

**Classifier: Support vector machine**

Features:

Age, Maximum diameter, Blood flow, Morphology, Uniformity

Parameters:

gamma: scale; kernel: rbf

**Single-modal models**

**Classifier: Support vector machine**

**Rad-based model:**

Features:

Shape_Elongation

Shape_Sphericity

Firstorder_Range

Gldm_DependenceEntropy

Glszm_SizeZoneNonUniformity

Glszm_SmallAreaEmphasis

Glszm_SmallAreaLowGrayLevelEmphasis

Glcm_ClusterShade

Glcm_Correlation

Gldm_DependenceNonUniformityNormalized

Glcm_Imc1

Glszm_LargeAreaHighGrayLevelEmphasis

Firstorder_MeanAbsoluteDeviation

Glszm_SizeZoneNonUniformityNormalized

Glszm_ZonePercentage

Firstorder_Maximum

Firstorder_RootMeanSquared

Glcm_Contrast

Glcm_DifferenceAverage

Glcm_Idmn

Glcm_Idn

Glszm_HighGrayLevelZoneEmphasis

Glszm_GrayLevelNonUniformityNormalized

Glszm_GrayLevelVariance

Glszm_SizeZoneNonUniformity

Glszm_SizeZoneNonUniformityNormalized

Glszm_ZonePercentage

Glszm_ZoneVariance

Firstorder_Energy

Firstorder_Kurtosis

Firstorder_Minimum

Firstorder_RootMeanSquared

Firstorder_TotalEnergy

Glcm_MaximumProbability

Glszm_ZoneEntropy

Parameters:

gamma: scale; kernel: linear

**DL-based model:**

Features:

Feature_5, Feature_33, Feature_43, Feature_57, Feature_60, Feature_90, Feature_107, Feature_109, Feature_127, Feature_133, Feature_158, Feature_160, Feature_168, Feature_174, Feature_189, Feature_191, Feature_196, Feature_199, Feature_208, Feature_224, Feature_235, Feature_236, Feature_242, Feature_247, Feature_258, Feature_270, Feature_277, Feature_286, Feature_305, Feature_312, Feature_313, Feature_319, Feature_322, Feature_329, Feature_338, Feature_395, Feature_400, Feature_402, Feature_410, Feature_415, Feature_420, Feature_424, Feature_446, Feature_447, Feature_448, Feature_453, Feature_466, Feature_472, Feature_485, Feature_488, Feature_500, Feature_508

Parameters:

gamma: scale; kernel: linear

**Pre-FMs**

**Classifier: Support vector machine**

**Pre-FM Clinic＋Rad:**

Features:

Age, Maximum diameter, Blood flow, Morphology, Uniformity

Firstorder_MeanAbsoluteDeviation

Firstorder_Range

Gldm_DependenceEntropy

Glszm_SizeZoneNonUniformity

Glszm_SmallAreaLowGrayLevelEmphasis

Glcm_Correlation

Firstorder_RootMeanSquared

Glszm_HighGrayLevelZoneEmphasis

Glszm_GrayLevelNonUniformity

Glszm_SizeZoneNonUniformity

Glszm_ZoneEntropy

Firstorder_Kurtosis

Glszm_GrayLevelNonUniformity

Glszm_ZonePercentage

Glcm_MaximumProbability

Gldm_GrayLevelNonUniformity

Parameters:

gamma: scale; kernel: linear

**Pre-FM Clinic＋DL:**

Features:

Age, Maximum diameter, Blood flow, Morphology, Uniformity, Feature_5, Feature_24, Feature_30, Feature_33, Feature_43, Feature_57, Feature_94, Feature_98, Feature_109, Feature_161, Feature_189, Feature_196, Feature_200, Feature_224, Feature_286, Feature_301, Feature_305, Feature_313, Feature_319, Feature_329, Feature_338, Feature_392, Feature_415, Feature_420, Feature_446, Feature_449, Feature_453, Feature_460, Feature_491, Feature_505, Feature_511

Parameters:

gamma: auto; kernel: linear

**Pre-FM Clinic＋Rad＋DL:**

Features: Age, Maximum diameter, Blood flow, Morphology, Uniformity

Shape_Elongation

Firstorder_MeanAbsoluteDeviation

Firstorder_Range

Gldm_DependenceEntropy

Glszm_SmallAreaLowGrayLevelEmphasis

Glcm_Correlation

Glszm_SizeZoneNonUniformityNormalized

Firstorder_RootMeanSquared

Glszm_ZoneEntropy

Glszm_HighGrayLevelZoneEmphasis

Glszm_LowGrayLevelZoneEmphasis

Glszm_SizeZoneNonUniformity

Glszm_ZoneEntropy

Firstorder_Kurtosis

Glszm_GrayLevelNonUniformity

Glcm_MaximumProbability

Gldm_GrayLevelNonUniformity

feature_33, feature_420, feature_449

Parameters:

gamma: scale; kernel: linear

**Post-FMs**

**Classifier: Classifier: Support vector machine**

**Post-FM Clinic+Rad:**

Model set: CM, Clinic＋Rad

Parameters:

gamma: scale; kernel: rbf

**Post-FM Clinic+DL:**

Model sets: CM, Clinic＋DL

Parameters:

gamma: scale; kernel: rbf

**Post-FM Clinic+Rad+DL**

Model sets: CM, Clinic＋Rad, Clinic＋DL

Parameters:

gamma: scale; kernel: rbf

**1.2 Code**

import os

from sklearn import preprocessing

from sklearn.model_selection import StratifiedKFold, cross_val_predict

from sklearn.svm import SVC

from sklearn.metrics import (

accuracy_score, precision_score, recall_score, f1_score,

roc_auc_score, confusion_matrix, matthews_corrcoef

)

from skopt.space import Real, Categorical

from skopt import BayesSearchCV

import pandas as pd

import numpy as np

import joblib

# import shap # If not used, it can be commented out

# Define SVM model and its parameter grid

models_params = {

'SVM': {

'model': SVC(probability=True),

'params': {

'C': Real(0.1, 200, prior='log-uniform'),

'kernel': Categorical(['linear', 'rbf', 'poly', 'sigmoid']),

'gamma': Categorical(['scale', 'auto'])

}

}

}

# Model selection mapping (only includes SVM)

all_selected = {0: 'SVM'}

def process_models(split_data, seed_range, csv_filename, model_name, model_info):

"""

Function to handle model training and evaluation, applicable only to SVM models.

Parameters:

- split_data: Data containing training and testing sets, format (X_train, y_train, X_test, y_test)

- seed_range: Range of random seeds for cross-validation

- csv_filename: Prefix for saving result files

- model_name: Name of the model (here, 'SVM')

- model_info: Model information, including model instance and parameter grid

"""

X_train, y_train, X_test, y_test = split_data

print(f"Processing {csv_filename} using model {model_name}")

param_results = []

performance_results = []

best_model_estimators = {}

for seed in seed_range:

print(f"Processing seed {seed}")

skf = StratifiedKFold(n_splits=5, shuffle=True, random_state=seed)

model = model_info['model']

param_grid = model_info['params']

bcv = BayesSearchCV(

model, param_grid, cv=skf, n_iter=30, scoring='roc_auc',

random_state=seed, verbose=0, n_jobs=-1 # Use all available CPU cores

)

bcv.fit(X_train, y_train)

# Save the best model for each seed

best_model_estimators[f"{model_name}_seed_{seed}"] = bcv.best_estimator_

# Save the best parameters

best_params = bcv.best_params_

best_params['Seed'] = seed

best_params['Model'] = model_name

param_results.append(best_params)

# Training set predictions

y_train_pred = cross_val_predict(bcv.best_estimator_, X_train, y_train, cv=skf, method='predict')

y_train_scores = cross_val_predict(

bcv.best_estimator_, X_train, y_train, cv=skf, method='predict_proba'

)[:, 1]

# Test set predictions

y_test_pred = bcv.best_estimator_.predict(X_test)

y_test_scores = bcv.best_estimator_.predict_proba(X_test)[:, 1]

# Calculate performance metrics

performance = {

'Seed': seed,

'Model': model_name,

'Accuracy': accuracy_score(y_test, y_test_pred),

'Precision': precision_score(y_test, y_test_pred, zero_division=0),

'Recall': recall_score(y_test, y_test_pred, zero_division=0),

'F1_Score': f1_score(y_test, y_test_pred, zero_division=0),

'ROC_AUC': roc_auc_score(y_test, y_test_scores),

'MCC': matthews_corrcoef(y_test, y_test_pred)

}

performance_results.append(performance)

# Convert results to DataFrame

param_results_df = pd.DataFrame(param_results)

performance_results_df = pd.DataFrame(performance_results)

# Save results to CSV

param_results_df.to_csv(f"{csv_filename}_params.csv", index=False)

performance_results_df.to_csv(f"{csv_filename}_performance.csv", index=False)

# Save the best models

joblib.dump(best_model_estimators, f"{csv_filename}_best_models.pkl")

print(f"Processing complete. Results saved as {csv_filename}_params.csv and {csv_filename}_performance.csv")

print(f"Best models saved as {csv_filename}_best_models.pkl")

return param_results_df, performance_results_df, best_model_estimators

# Example call (provide split_data and other parameters as per your context)

# split_data = (X_train, y_train, X_test, y_test)

# seed_range = range(42, 52) # For example, using 10 different seeds

# csv_filename = "svm_results"

# model_name = "SVM"

# model_info = models_params['SVM']

# process_models(split_data, seed_range, csv_filename, model_name, model_info)

# Supplementary Figures and Tables

## Supplementary Figures


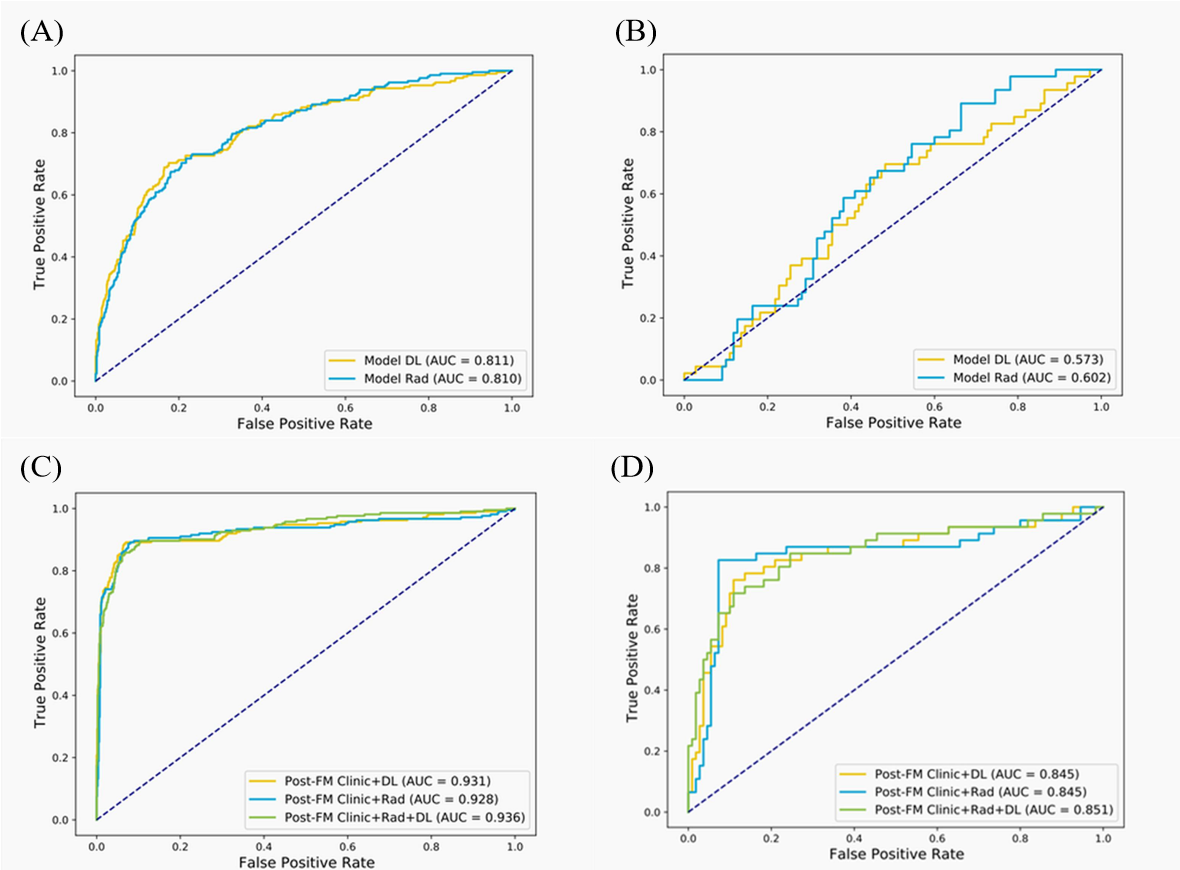


**Supplementary Figure 1.** Receiver operating characteristic (ROC) curves for the unimodal model in the training cohort (A) and the external validation cohort (B). ROC curves for the post-fusion model in the training cohort (C) and the external validation cohort (D).


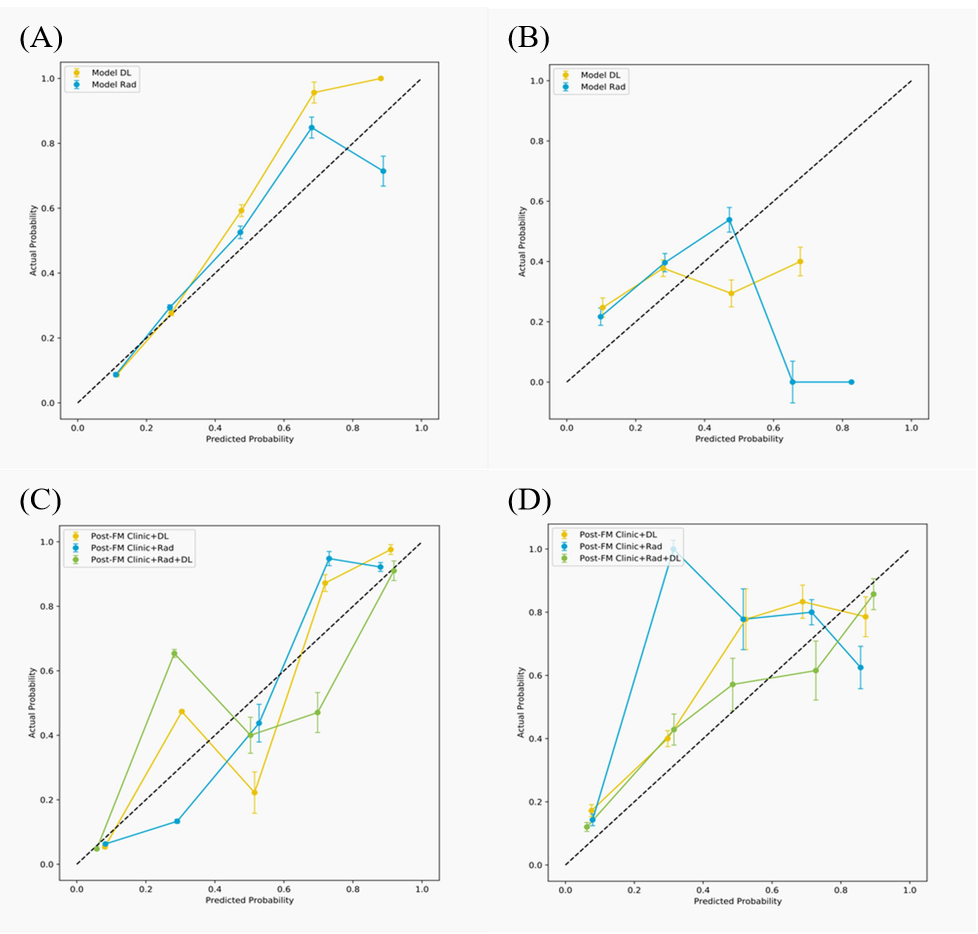


**Supplementary Figure 2.** Calibration curves of the unimodal model in the training cohort (A) and the external validation cohort (B). Calibration curves of the post-fusion model in the training cohort (C) and the external validation cohort (D).


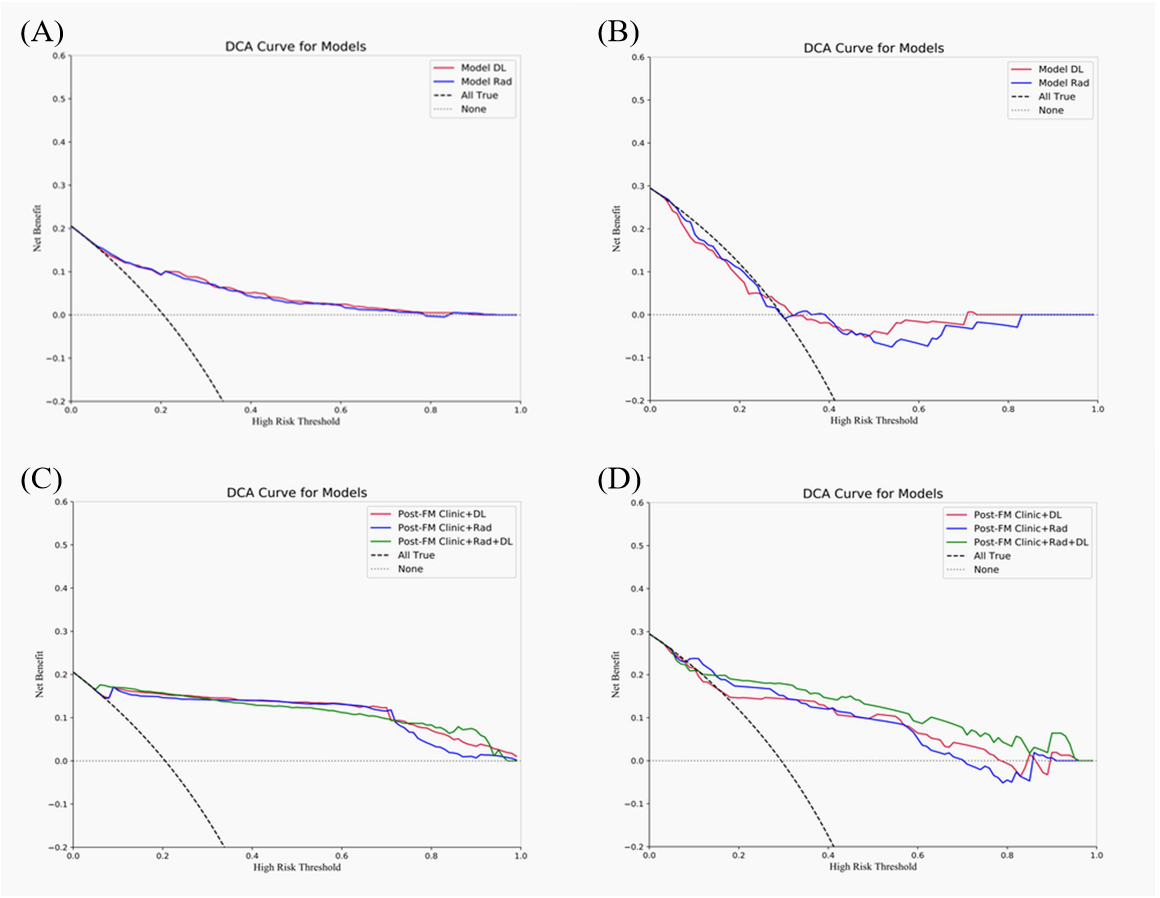


**Supplementary Figure 3.** Decision curve analysis (DCA) of the unimodal model in the training cohort (A) and the external validation cohort (B). DCA of the post-fusion model in the training cohort (C) and the external validation cohort (D).

# 2.2 Supplementary Tables

Supplementary Table 1. The Names and Descriptions of Ultrasound Semantic Features

| **Features** | **Description** |
| --- | --- |
| Maximum diameter（cm） | Diameter measured at the largest cross-section of the tumor on ultrasound. |
| Blood flow (0-1/2-3) | Grade 0: no blood flow signal is found within the mass; Grade 1: a small amount of blood flow, with 1 or 2 punctate or thin rod-shaped tumor vessels visible, and the rod-shaped blood flow is no more than 1/2 of the diameter of the lesion; Grade 2: a moderate amount of blood flow, with 3 or 4 punctate vessels or a longer vessel penetrating the lesion, which may be close to or longer than the radius of the mass; and Grade 3: a large amount of blood flow, with ≥5 punctate vessels or 2 longer vessels visible. (Derived from Adler blood flow grading) |
| Shape (regular/irregular) | Regular: the edges of the shape are smooth and neat, and the overall shape is round or oval; Irregular: the edges of the shape are rough, not smooth, or present an uneven appearance, and the shape is not symmetrical. |
| Boundary (clear/blurred) | Sharp margins indicates that the tumor margins are 100% clear. |
| Internal echoes (uniform/uneven) | Uniform internal echoes: internal echoes are smooth and consistent, with no obvious bright spots or dark areas; Uneven internal echoes: internal echoes show varying degrees of bright spots, dark areas, or structural complexity. |

Supplementary Table 2. The Names and Descriptions of Ultrasound Semantic Features

| Type of Mass | Primary cohort  (n = 516) | External validation  Cohort (n = 78) | Total  (n = 227) |
| --- | --- | --- | --- |
| Benign | 410 | 55 | 465 |
| Haemangioma | 47 | 6 | 53 |
| Glomangioma | 9 | 1 | 10 |
| Lipoma | 72 | 10 | 82 |
| epidermoid cyst | 40 | 3 | 43 |
| Schwannoma | 45 | 6 | 51 |
| Tenosynovial cyst | 32 | 1 | 33 |
| Tenosynovial giant cell tumour | 43 | 0 | 43 |
| Synovial cyst | 40 | 0 | 40 |
| Fibroma | 24 | 13 | 37 |
| Fibrous histiocytoma | 6 | 0 | 6 |
| Nodular fasciitis | 5 | 0 | 5 |
| Calcified epithelioma | 5 | 3 | 8 |
| Myxoma | 5 | 1 | 6 |
| Leiomyoma | 5 | 0 | 5 |
| Osteochondroma | 4 | 2 | 6 |
| Seborrheic keratosis | 0 | 3 | 3 |
| Granuloma | 1 | 2 | 3 |
| Proliferative myositis | 2 | 1 | 3 |
| Pigmented villonodular synovitis | 2 | 0 | 2 |
| Neuroma | 3 | 1 | 4 |
| Trichoblastoma | 1 | 0 | 1 |
| Cyst | 19 | 2 | 21 |
| Intermediate | 11 | 0 | 11 |
| Atypical lipomatous tumour | 4 | 0 | 4 |
| Atypical neurofibroma | 2 | 0 | 2 |
| Desmoid-type fibromatosis | 4 | 0 | 4 |
| Angiomatoid fibrous histiocytoma | 1 | 0 | 1 |
| Malignant | 95 | 23 | 118 |
| Myeloma | 1 | 0 | 1 |
| Sarcoma | 81 | 8 | 89 |
| Metastases | 8 | 10 | 18 |
| Melanoma | 0 | 2 | 2 |
| Basal cell carcinoma | 0 | 2 | 2 |
| Lymphoma | 2 | 0 | 2 |
| Epithelial-myoepithelial carcinoma | 2 | 1 | 3 |
| Peripheral nerve sheath tumour | 1 | 0 | 1 |

Supplementary Table 3. Univariate and Multivariate Analyses of Clinical Variables

| Variable | Univariate analysis | | |  | Multivariate analysis | | |
| --- | --- | --- | --- | --- | --- | --- | --- |
|  | OR | 95%CI | P |  | OR | 95%CI | P |
| Gender | 0.89 | 0.58-1.36 | 0.59 |  |  |  |  |
| Blood flow | 9.86 | 6.10-15.92 | <0.001 |  | 5.07 | 2.83-9.19 | <0.001 |
| Boundary | 5.19 | 3.16-8.52 | <0.001 |  | 1.60 | 0.82-3.12 | 0.168 |
| Morphology | 9.78 | 5.80-16.50 | <0.001 |  | 5.13 | 2.79-9.72 | <0.001 |
| Uniformity | 14.4 | 5.20-39.91 | <0.001 |  | 5.50 | 2.00-19.71 | 0.003 |
| Age | 1.04 | 1.02-1.05 | <0.001 |  | 1.04 | 1.02-1.05 | <0.001 |
| Maximum diameter | 1.32 | 1.23-1.42 | <0.001 |  | 1.21 | 1.11-1.33 | <0.001 |

95%CI= 95% confidence interval; OR= odds ratio.

Supplementary Table 4. Performance of DL-based models and Rad-based models in the training and external validation cohort

| Group | Model | AUC | Sensitivity (%) | Specificity (%) | PPV (%) | NPV (%) | Accuracy (%) |
| --- | --- | --- | --- | --- | --- | --- | --- |
| TC | Model DL | 0.751(0.741-0.760) | 81.3 | 70.3 | 98.7 | 12.3 | 80.9 |
|  | Model Rad | 0.757(0.749-0.765) | 81.9 | 56.1 | 96.5 | 17.5 | 80.2 |
| EVC | Model DL | 0.573(0.547-0.598) | 70.9 | 40.0 | 97.3 | 4.3 | 69.9 |
|  | Model Rad | 0.602(0.576-0.629) | 69.4 | 16.7 | 90.9 | 4.3 | 65.4 |

AUC = area under receiver operating characteristic curve; DL = deep learning; EVC = external validation cohort. NPV = negative predictive value; PPV = positive predictive value; Rad = radiomics; TC = training cohort.

Supplementary Table 5. Performance of Post Fusion Models (Post-FM) in the training and external validation cohort

| Group | Model | AUC | Sensitivity (%) | Specificity (%) | PPV (%) | NPV (%) | Accuracy (%) |
| --- | --- | --- | --- | --- | --- | --- | --- |
| TC | Post-FM Clinic + Rad | 0.881(0.864-0.899) | 92.9 | 84.0 | 96.5 | 71.7 | 91.4 |
|  | Post-FM Clinic + DL | 0.888(0.871-0.905) | 92.7 | 84.7 | 96.7 | 70.8 | 91.4 |
|  | Post-FM CRDL | 0.921(0.902-0.940) | 92.8 | 83.0 | 96.2 | 71.2 | 91.1 |
| EVC | Post-FM Clinic + Rad | 0.845(0.804-0.885) | 83.6 | 76.5 | 92.7 | 56.5 | 82.1 |
|  | Post-FM Clinic + DL | 0.945(0.803-0.887) | 83.2 | 80.6 | 94.5 | 54.3 | 82.7 |
|  | Post-FM CRDL | 0.851(0.800-0.902) | 85.7 | 78.4 | 92.7 | 63.0 | 84.0 |

AUC = area under receiver operating characteristic curve; CRDL = clinical and radiomics and deep learning; DL = deep learning; EVC = external validation cohort; NPV = negative predictive value; Post-FM= post-fusion model; PPV = positive predictive value; Rad = radiomics; TC = training cohort.

Supplementary Table 6. Comparison of Diagnostic Performance Between Post-Fusion Models and Single-Mode Models

| Model vs. Model | P Value | |
| --- | --- | --- |
| PC | |  |
| Model Rad vs. Model DL | | 0.972 |
| Post-FM Clinic + Rad vs. Post-FM Clinic + DL | | 0.775 |
| Post-FM Clinic + Rad vs. Post-FM CRDL | | 0.481 |
| Post-FM Clinic + DL vs. Post-FM CRDL | | 0.612 |
| EVC | | 0.563 |
| Model Rad vs. Model DL | |  |
| Post-FM Clinic + Rad vs. Post-FM Clinic + DL | | 0.991 |
| Post-FM Clinic + Rad vs. Post-FM CRDL | | 0.855 |
| Post-FM Clinic + DL vs. Post-FM CRDL | 0.860 | |

CRDL = clinical and radiomics and deep learning; DL = deep learning; EVC = external validation cohort; PC = primary cohort; Post-FM= post-fusion model; Rad = radiomics.
